# Tachycardiomyopathy

**Published:** 2002-10-01

**Authors:** Yuji Nakazato

**Affiliations:** Department of Cardiology, Juntendo University School of Medicine, Tokyo, Japan

## Introduction

Sustained chronic tachyarrhythmias often cause a deterioration of cardiac function known as tachycardia-induced cardiomyopathy or tachycardiomyopathy [1]. It has been recognized that the tachycardiomyopathy occurs in experimental models2 and also in patients with supraventricular or ventricular tachycarrhythmias [3-6]. Generally, cardiac function will recover if drug or catheter ablation therapy is successfully performed. Phillips and Levine [7] initially presented this concept in 1949 for the relationship between rapid atrial fibrillation and reversible left ventricular (LV) failure. However, it might be difficult to define its cause and effect relationship when cardiomyopathy and tachycardia are identified simultaneously. The reversibility of LV dysfunction may often be variable and the precise mechanisms of the pathophysiological features of this phenomenon should be elucidated. Although excellent and comprehensive review articles by Fenelon G, et al [8] and Shinbane JS et al [9] regarding these topics have already been published, I will firstly present a typical case and then review the basic and clinical characteristics of tachycardiomyopathy in this article.

## Case Presentation [10]

A 13-year-old girl was admitted with a chief complaint of orthopnea. She had been born with the congenital anomalies of spina bifida and common cloacae. At birth, an artificial anal and urinal plasties had been performed. At the age of 11 her urinary and anal tracts had been surgically separated. After the operation she has been doing well, but at the age of 13, orthopnea and edema of the lower extremities was noticed. She was referred to pediatrics and diagnosed as having congestive heart failure with atrial tachycardia. Digitalis, verapamil, and a small dose of metoprolol were not effective in controlling her arrhythmia, so she was referred to our department for further evaluation and treatment.

**Figure 1:**
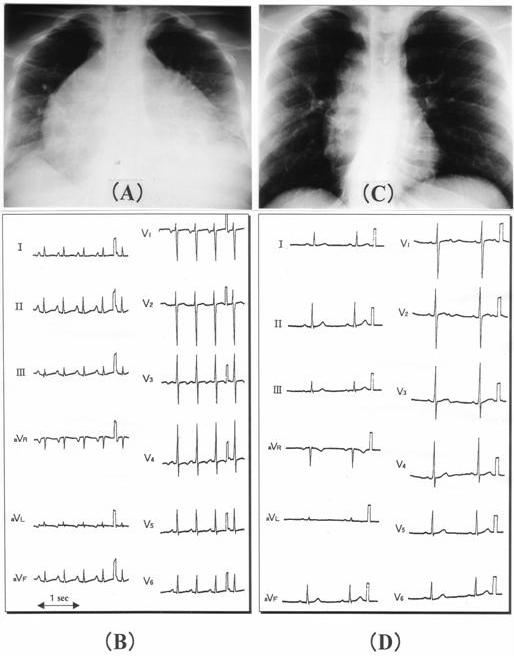
(A) Chest X-P on admission. Cardiothrocic ratio was 77%. (B) ECG with ectopic atrial tachycardia (150 bpm). (C) Chest X-P after pilsicainide administration. CTR was 46%. (D) ECG with sinus rhythm (60 bpm).

Physical examination indicated a small build (body height 112cm, weight 26kg) with underdevelopment of the lower extremities. Chest X-ray revealed marked cardiomegaly with a cardio-thoracic ratio of 77 % (Figure 1A). On echocardiography, the left ventricular ejection fraction (LVEF) and the diastolic dimension were 0.21 and 51 mm, respectively. Her ECG showed atrial tachycardia with a heart rate of 150 beats per minute (bpm) and an upright P wave configuration in the limb leads of II,III, and aVf (Figure 1B).

At first we sought to perform an electrophysiological study, but the venous approach for catheterization was quite difficult since the development of her bilateral femoral vein had been hindered by the operation for her congenital genito-urinal abnormality. Furthermore, the patient and her family were reluctant to accept invasive examination or treatment. Thereafter, we decided to prescribe pilsicainide at an initial dose of 25 mg, and within 2 hours her ectopic atrial tachycardia was converted to a sinus rhythm. It was maintained by a daily dose of 75 mg pilsicainide from that day onward. After 3 months, chest X-rays showed an improvement of the cardiothoracic ratio to 46% (Figure 1C), while her sinus rhythm was maintained using the same prescription (Figure 1D). On echocardiography, the LV EF improved to 0.62 and the diastolic dimension decreased to 37 mm. She has been well in the four years since, and repeated Holter recordings have indicated the successful maintenance of sinus rhythm.

## Definition and Classification

Coined by Gallagher JJ [11] , the term tachycardia-induced cardiomyopathy or tachycardiomyopathy refers to impairment in LV function secondary to chronic tachycardia, which is partially or completely reversible after normalization of heart rate and/or rhythm irregularity. Later, Brugada P and Andries E [12] defined that a "tachycardiomyopathy" is an abnormality of systolic or diastolic function of the heart, or both, usually resulting in heart dilatation and ultimately in heart failure caused by a high and/or irregular ventricular rate. This high and/or irregular ventricular rate may result from any type of cardiac arrhythmia.

Fenelon et al [8] further classified tachycardiomyopathy into two categories. They are "pure type" and "impure type" tachycardiomyopathy. In the former, chronic tachycardia causes LV dysfunction in a normal heart and completely recovers after termination of the tachycardia. In the latter, such a condition occurs in patients with structural heart diseases, and the cardiac dysfunction may only recover incompletely after termination of the tachycardia. The incomplete recovery of LV function might be the result of long-term tachycardia inducing irreversible myocardial injury.

## Mechanism of Tachycardiomyopathy

There is no definite answer to the precise mechanism of tachycardiomyopathy in the clinical setting [8,9]. Several experimental studies have however been helpful in understanding the pathophysiology of tachycardiomyopathy.

### Basic mechanisms

Tomita et al [13] investigated three groups of pigs:1)rapid left atrial pacing for 3 weeks, 2)with a 4-week recovery period, and a 3)sham-group. In their experimental models, a continuous pacing rate of more than 240 beats/min for 3 weeks provoked the decrease of cardiac output, dilatation of the left ventricle, reduced systolic function and diastolic dysfunction, such as increased LV end-diastolic pressure, as well as neurohumoral abnormalities similar to human dilated cardiomyopathy. They found that LV wall thickness was  reduced, but the ventricular mass itself did not change. After termination of the pacing, systolic function recovered ,but diastolic function remained abnormal. Thus, the severity of LV dysfunction was related to the duration of tachyarrhythmias.

In chronic-paced animal models, the response to beta-adrenergic stimulation is known to be blunted. This may also be related to a decrease in the density of the beta-adrenergic receptors [14,15]. These facts are often confirmed in clinical situations. Catecholamine concentrations in patients with heart failure are increased at rest and during low-level exercises, and attenuated during high-level exercises [16]. Such inappropriate adrenergic responses may facilitate the development of tachycardiomyopathy and therefore, the efficacy of adequate beta-blocker therapy may be expected in treating tachycardiomyopathy [17]. Actually, beta-blockers had favorable effects in patients with dilated cardiomyopathy [18,19]. Other neurohumoral activation has also been noted with a marked elevation of plasma natriuretic peptides, rennin activity and aldosterone levels [20].

Regarding other cellular functions, chronic SVT induced cardiomyopathy in swine was associated with decreased Na-K ATPase activity and glycoside receptor density and affinity [21,22]. Spinale et al reported that the contractile function in isolated myocytes is decreased due to the blunted response to any extracellular calcium overloads [23]. According to He JQ et al [24], cellular remodeling in heart failure results in the decreased density of T-tubules and L-type Ca2+ channels, which contribute to abnormal EC coupling. It may contribute to the reduction of contractility in tachycardia-induced cardiomyopathy. Furthermore, Williams R et al [25] referred to a relationship between myocardial protein gene expression and decreased LV contractility in their canine model.

A recent experimental study indicated that antioxidant vitamins such as beta-carotene, ascorbic acid and alpha-tocopherol reduced tissue oxidative stress in congestive heart failure and attenuated the associated cardiac dysfunction in a rabbit model. In addition, they also attenuated beta-receptor down regulation and sympathetic nerve terminal abnormalities. Therefore, it was emphasized that antioxidant therapy may be an efficacious method in treating human congestive heart failure [26].

In a pig model of tachycardiomyopathy, Spinale et al [27] observed a reduced myocardial blood flow reserve, particularly in the subendocardium and a decrease in endocardial/epicardial flow ratio. No significant morphological changes in the coronary vasculature were observed. This suggests that the vascular structural changes were not responsible for the abnormalities in myocardial blood flow. The LV dysfunction and myocyte injury linked to tachycardiomyopathy are thought to be associated with reduced myocardial blood flow. Early recovery from supraventricular tachycardiomyopathy resulted in hypertrophy with normal myocardial blood flow at rest, but significantly reduced coronary reserve. However, these blood flow abnormalities in tachycardiomyopathy have not sufficiently been evaluated in clinical series.

In relation to the basic mechanisms of myocardial cell hypertrophy, Clemo et al [28] tested the hypothesis that cellular hypertrophy in congestive heart failure modulates mechanosensitive channels. In canine models, swelling-activated inward rectifying cation current (I circ, swell) is persistently activated in tachycardiomyopathy. It is likely to be activated in multiple forms of cardiac hypertrophy and failure and may contribute to dysrythmias and altered contractile function. They suggested that I circ, swell may represent a novel target for therapeutic interventions.

A detailed summary of the functional and neurohumoral spectra of experimental tachycardiomyopathy is described by Fenelon et al [8]. in their review.

In addition, recent studies elucidated that the progression of tachycardiomyopathy to heart failure facilitates ionic channel remodeling and it may contribute the generation of ventricular tachyarrhythmias. There have been many studies related to the ionic remodeling (K+ and Ca2+) of ventricular myocytes in experimental animals by rapid-pacing induced heart failure [29,30]. A reduction in the density of transient potassium outward current(Ito) is the most consistent ionic current change in cardiac failure. The density of Ito is known to be variable regionally and transmurally, and it was reduced differentially in heart failure [31]. Tomaselli GF et al  [32]indicated that the down-regulation of Ito may generate repolarization abnormalities and cause increased electrical instability of the failing heart. A recent study also pointed out the down-regulation of two components of the delayed rectifier potassium current (Ikr and Iks) in the pacing-induced heart failure rabbit model [33]. It may prolong the action potential at physiological cycle lengths and therefore contribute to arrhythmogenesis in heart failure. Han W, et al [34] studied ionic remodeling of cardiac purkinje cells(PCs) isolated from control and pacing-induced heart failure dogs. Their results showed that remodeling of K+ and Ca2+ currents occurs in PCs of tachycardia-induced heart failure dogs. Ik1 and Ito density were significantly smaller in congestive heart failure PCS and cause a reduction of the repolarization reserve. They concluded that the results may explain the ventricular arrhythmogenesis, particulary related to triggered activity in PCs, in patients with heart failure.

### Clinical mechanisms

In clinical situations, tachycardiomyopathy is induced by various supraventricular and ventricular arrhythmias. Particularly in childhood, ectopic atrial tachycardia (EAT), a permanent form of junctional reciprocating tachycardia (PJRT) are most prevalently observed, which they are often incessant and refractory to antiarrhythmic drugs, and this commonly results in tachycardiomyopathy [35].

Human tachycardiomyopathy is considered to be similar to the mechanisms of most experimental models. A small number of studies suggest a hemodynamical changes in patients with tachycardiomyopathy [36]. They are reduced LVEF, increased end-diastolic and end-systolic volumes, and increased end-diastolic and pulmonary artery pressures. Termination of the tachycardia lead to an improvement of clinical symptoms, an increase of EF and a marked decrease of end-systolic volumes. Concentric LV hypertrophy following recovery from tachycardiomyopathy was not noted. Usually, the degree of LV dysfunction is not always related to the tachycardia duration or rate [1]. Thease reasons are why the determination of the onset is quite difficult, since the patients without underlying diseases are asymptomatic until congestive heart failure occurs. On the other hand, patients with underlying heart disease are more likely to progress toward heart failure.

Paelinck B et al  [37] reported an evaluation method of myocardial contractile reserve in dilated cardiomyopathy with atrial fibrillation by using low-dose dobutamine echocardiography. They investigated using a wall motion score index, which was obtained by a summation of the individual wall motion scores of 16 segments of the left ventricle at baseline and after infusion of low-dose dobutamine as well as before and after cardioversion . The patients who showed a marked increase of LVEF at low-dose dobutamine and normalized EF at the follow-up were considered to have cardiomyopathy. These patient's wall motion score index had significantly improved, both at low-dose dobutamine and at the follow-up. On the contrary, no or low responders to dobutamine and wall motion score index were diagnosed as having dilated cardiomyopathy. It was suggested that low-dose dobutamine echocardiography predicts the improvement in LVEF after restoration of the sinus rhythm and is thus available in the identification of tachycardiomyopathy.

Other factors related to LV function; age, underlying heart disease, drugs, irregurality of ventricular rhythm, short atrioventricular interval of less than 100ms are emphasized, yet remain to be determined [8].

Regarding the mechanism of functional mitral regurgitation in tachycardiomyopathy, Timek TA et al [38] studied the valvular and ventricular 3D geometric perturbations associated with MR in an ovine model of tachycardia-induced cardiomyopathy. According to the findings, the result of mitral annular dilatation and separation of the leaflet hinge points are the primary cause, which then led to incomplete leaflet coaptation and valve incompetence. Altered subvalvular leaflet tethering did not appear to play a major role in the pathogenesis of MR.

## Clinical Diagnosis

A definite diagnosis of tachycardiomyopathy is sometimes difficult. A correct diagnosis can only be made by a normalization or improvement of the impairment of LV function after control of the tachyarrhythmias. However, it is also a fact that tachyarrhythmia control does not always bring about the improvement of LV function in patients with tachycardiomyopathy, and may simply reflect the irreversible stage of tachycardiomyopathy and complicate the diagnosis. Thus, no specific methods are available to identify the presence of tachycardiomyopathy at present. Therefore, it is important to suspect the existence of this condition from its history and clinical findings.

Fenelon et al  [8] proposed the following criteria for the diagnosis of tachycardiomyopathy; in patients presenting with; 1) dilatation of the heart or heart failure, 2) Chronic or very frequent cardiac arrhythmias, including incessant supraventricular tachycardia, atrial fibrillation or flutter and incessant ventricular tachycardia. They further pointed out that if chronic tachycardia continued more than 10-15% of the day, with an atrial rate of more than 150% of that predicted for age, tachycardiomyopathy ensues.

## Treatment

The basic concept for the treatment of tachycardiomyopathy is in controling the heart rate. The appropriate heart rate is beneficial for patients with tachycardia-induced heart failure. The usual approaches for tachycardiomyopathy are by both pharmacological and non-pharmacological treatments.

In pharmacological therapy, drug choice depends on the underlying arrhythmias. In supraventricular tachycardia, digitalis or a Ca2+ antagonist (verapamil) are the common drugs of choice. Hayano M, et al [39] reported a successful treatment of ectopic atrial tachycardiomyopathy by digitalis and verapamil. After gaining control of the heart rate, the LV diastolic dimension and cardiothoracic ratio were improved. They emphasized that initial medical treatment is preferable in the absence of a clinical emergency.

Usually, class I anti-arrhythmic drugs have a negative inotropic effect and therefore, the clinical use of these drugs for tachycardiomyopathy should be carefully decided according to the patient's background. The case presented  here also had ectopic atrial tachycardiamyopathy, which was successfully treated with pilsicainide. This drug is a class Ic drug with a strong sodium channel blocking action and has a relatively short half-life compared to other Ic agents. In addition, a single oral dose is often effective for terminating supraventricular arrhythmias. For these reasons we cautiously administrated a single oral dose of pilsicainide (25mg), and it eventually terminated the EAT. Sinus rhythm was maintained at a dose of 75 mg/day and dramatic improvement of cardiac function was observed. Thus, if for some reason, catheter ablation cannot be performed as the first-line of therapy, class Ic drugs, including pilsicainide are an available option for the treatment of EAT-induced cardiomyopathy, provided that the patient is carefully observed.

In pediatric cases, EAT or PJRT is often incessant and commonly results in tachycardiomyopathy.  Amiodarone is the only available drug for patients with impaired LV function and tachyarrhythmias.40 In pediatric cases, EAT or PJRT is often incessant and commonly results in tachycardiomyopathy. Chen R.P-C [41]. reported a case of PJRT which was successfully treated with amiodarone during the neonatal period. After 6 weeks systolic function improved. Complete resolution of ventricular tachycardia-induced cardiomyopathy by oral amiodarone in a young boy has also been reported [42]. Treatment with other class III drugs including dofetilide, sotalol, ibutilide or azimilide may be another option. These class III drugs prolong the action potential duration and may enhance the inward calcium reflux resulting in increased contractility and are a favorable choice for tachycardiomyopathy if adverse effects are avoided. However, medical treatment is not always effective and safe, besides, several available drugs are ineffective in such cases. Therefore, a non-pharmacological approach is the next choice in the treatment.

## Non-pharmacological approach

### Catheter ablation

Since the introduction of radiofrequency (RF) ablation, the treatment of tachyarrhythmias has been safely performed and favorable results have been obtained. Simultaneously, it has been proven that the cure or control of tachyarrhythmia could improve LV function and heart failure in patitents with tachycardiomyopathy. Targeted arrhythmias are wide ranging, including EAT [43], PSVT or  [44-47] PJRT atrial fibrillation/flutter [3,48] frequent isolated premature ventricular extrasystole [49], and ventricular tachycardia  [51-53]. In most of these reports, cardiac function improved or normalized after successful RF ablation. These facts lead us to believe that catheter ablation in patients with tachycardiomyopathy should be performed as the first-line therapy as soon as possible.

RF ablation can even be used successfully even in a cardiac transplant patient with atial tachycardia-induced cardiomyopathy [54].

### Surgical therapy

Similary, a surgical treatment is also effective for patients who received operations to cure the tachyarrhythmias. Giorgi LV et al [55] reported the surgical treatment of an 11 year-old-girl with focal atrial tachycardia and congestive heart failure which resulted in the improvement of LV function. Cruz FES et al [4] reported the experience of surgical treatment of PSVT induced cardiomyopathy in 8 of 17 patients. After an average follow-up of 21.6 months, EF increased from 36% to 59%, LVDd decreased from 56 mm to 49 mm. They acknowledged the significance of surgery in such patients. Rabbani et al [56] reported a case of DCM with EAT which was treated surgiclly. The patient received surgical cryoablation and LV function was improved 1 month after the operation. If catheter ablation cannot be performed as the first-line therapy for some reason, surgical therapy is an available option for the treatment of tachycardiomyopathy.

## Prognosis

Generally, if tachyarrhythmias are properly treated, cardiac function will recover. In bacic study, the recovery of LV systolic function requires about 2 weeks from the termination of pacing. However, in clinical situations, this may be quite variable both spatially and temporally because the length of tachycardia and underlying heart diseases are different in each patient.

## Conclusion

The diagnosis of tachycardia-induced cardiomyopathy is not always simple, but we should start therapy if the patient suffers from congestive heart failure with tachyarrhythmias. This is because LV dysfunction may be recover after adequate treatment and it is the only way to make a definite diagnosis of tachycardiomyopathy.
